# Anterior Chamber Angle Assessment Techniques: A Review

**DOI:** 10.3390/jcm9123814

**Published:** 2020-11-25

**Authors:** Ivano Riva, Eleonora Micheletti, Francesco Oddone, Carlo Bruttini, Silvia Montescani, Giovanni De Angelis, Luigi Rovati, Robert N. Weinreb, Luciano Quaranta

**Affiliations:** 1 IRCCS-Fondazione Bietti, 00198 Rome, Italy; ivano.riva@virgilio.it (I.R.); oddonef@gmail.com (F.O.); 2 Department of Surgical and Clinical, Diagnostic and Pediatric Sciences, Section of Ophthalmology, University of Pavia, IRCCS Fondazione Policlinico San Matteo, 27100 Pavia, Italy; eleonora.micheletti@gmail.com (E.M.); carlo.bruttini@hotmail.it (C.B.); silvia.montescani@gmail.com (S.M.); 3 Department of Surgical and Clinical, Diagnostic and Pediatric Sciences, Section of Ophthalmology, University of Pavia, 27100 Pavia, Italy; gianni.deangelis@iol.it; 4 Department of Engineering “Enzo Ferrari”, University of Modena and Reggio Emilia, 41125 Modena, Italy; luigi.rovati@unimore.it; 5 Hamilton Glaucoma Center, Shiley Eye Center and Department of Ophthalmology, University of California, La Jolla, San Diego, CA 92093, USA; rweinreb@health.ucsd.edu

**Keywords:** diagnosis, trabecular meshwork, anterior chamber angle, iridocorneal angle, angle closure glaucoma

## Abstract

Assessment of the anterior chamber angle (ACA) is an essential part of the ophthalmological examination. It is intrinsically related to the diagnosis and treatment of glaucoma and has a role in its prevention. Although slit-lamp gonioscopy is considered the gold-standard technique for ACA evaluation, its poor reproducibility and the long learning curve are well-known shortcomings. Several new imaging techniques for angle evaluation have been developed in the recent years. However, whether these instruments may replace or not gonioscopy in everyday clinical practice remains unclear. This review summarizes the last findings in ACA evaluation, focusing on new instruments and their application to the clinical practice. Special attention will be given to the comparison between these new techniques and traditional slit-lamp gonioscopy. Whereas ultrasound biomicroscopy and anterior segment optical coherence tomography provide quantitative measurements of the anterior segment’s structures, new gonio-photographic systems allow for a qualitative assessment of angle findings, similarly to gonioscopy. Recently developed deep learning algorithms provide an automated classification of angle images, aiding physicians in taking faster and more efficient decisions. Despite new imaging techniques made analysis of the ACA more objective and practical, the ideal method for ACA evaluation has still to be determined.

## 1. Introduction

Evaluation of the anterior chamber angle (ACA) is an essential part of the ophthalmological examination, instrumental to achieve pertinent relevant information on glaucoma patients as well as on non-glaucomatous subjects [1]. In patients with glaucoma or glaucoma suspicion, a careful assessment of the ACA should always be performed, allowing direct visualization of the main structures cause of the aqueous humor drainage, directly affecting intraocular pressure [1,2]. Several findings may be associated with an impaired aqueous humor outflow, among them abnormal iris insertion, abnormal pigmentation of the trabecular meshwork (TM), presence of synechiae, blood in the Schlemm’s canal (SC), angle recession, abnormal blood vessels in the angle, evidence of anterior segment dysgenesis, and other abnormalities [3].

Although less prevalent worldwide than primary open-angle glaucoma, primary angle-closure glaucoma prevalence has been estimated to be 22 million people in 2013, with the highest numbers in people of Asian ancestry [4]. In China, it was estimated that 9 million people have a significant angle closure and more than 28 million people have an anatomic trait predisposing to primary angle closure glaucoma (an “occludable” drainage angle) [5]. Despite these data, the definition of “occludable angle” is in fact unclear both in the literature and in the authoritative clinical guidelines on glaucoma. According to the most widely used classification, an occludable angle is defined as an angle in which the TM is not gonioscopically identifiable in more than 90° of angle circumference [6,7]. However, in 2004 Foster et al. reported that this definition should be reconsidered, and suggested that angles between 10° and 20° should be defined as angles with a “probable” and a “possible” risk of closure, respectively [8]. It should be noted that the management of occludable angles is also poorly defined. In a recent randomized controlled trial on patients with primary angle closure suspicion [9], prophylactic peripheral iriditomy had little effect on preventing the progression towards primary angle closure. These results may be justified, at least partially, if the low rate of progression from primary angle closure suspicion to angle-closure glaucoma found in this study is properly taken into account.

It has been demonstrated that without an appropriate gonioscopic evaluation, the vast majority of chronic angle closure varieties may be mistaken for open-angle glaucoma [10,11]. In this respect, Varma et al. found that approximately 10% of patients diagnosed with primary open-angle glaucoma were actually affected by angle closure glaucoma [12]. Slit-lamp gonioscopy is still the clinical reference standard for the assessment of the irido-corneal angle, playing a fundamental role in the distinction between open and closed angle glaucoma, and consequently in the determination of the future disease management [10,11]. However, gonioscopy is performed in approximately half of the ophthalmological visits, and assessment of the ACA during the follow-up is poor, even among glaucoma specialists [13]. It has been demonstrated that the repeatability of gonioscopy is higher when the examination is performed by highly experienced vs. novice personnel, as the regular practice and the retraining are likely to improve and maintain the performance [14]. This has been found in a collaborative care glaucoma clinic, where a “fair to moderate” agreement in gonioscopy was achieved between experienced optometrists and glaucoma specialists [15].

Several imaging technologies have been developed in recent years, to make the evaluation of the ACA more quantitative and practical: ultrasound biomicroscopy (UBM), gonio-photographic systems (GPS), limbal anterior chamber depth measurement (LACDM), known also as Van Herick test, scanning peripheral anterior chamber depth analyzer (SPAC), Scheimpflug photography (SP), and anterior segment optical coherence tomography (AS-OCT), which may provide a more objective evaluation of the ACA structures [16]. Deep learning algorithms have been recently introduced as well, to automate the analysis of angle images [17].

**Figure 1:**
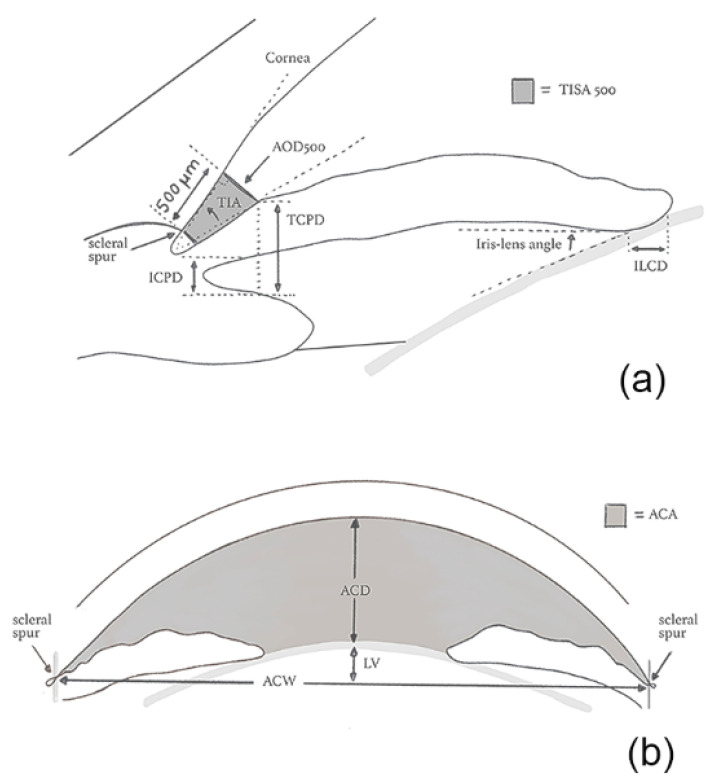
Quantitative parameters of the irido-corneal angle (**a**) and of the anterior chamber (**b**). ACA: Anterior chamber angle; ACD: Anterior chamber depth; ACW: Anterior chamber width; AOD: Angle opening distance; ICPD: Iris-ciliary process distance; ILCD: Iris-lens contact distance; LV: Lens vault; TIA: Trabecular-iris angle; TISA: Trabecular iris space area; TCPD: Trabecular-ciliary process distance.

A variety of quantitative parameters describing the anterior segment anatomic features have been proposed, namely: angle opening distance (AOD), angle recess area (ARA), trabecular-iris angle (TIA), trabecular-iris space area (TISA), trabecular-ciliary process distance (TCPD), anterior chamber width (ACW), peripheral anterior chamber depth (ACD), anterior chamber volume (ACV), and Van Herick’s grading (VHG) (Figure 1).

The aim of this paper is to give an overview of the emerging techniques for the evaluation of the ACA, focusing on their potential role in clinical practice, especially when compared to traditional techniques.

## 2. Contact Techniques

### 2.1. Gonioscopy

**Figure 2:**
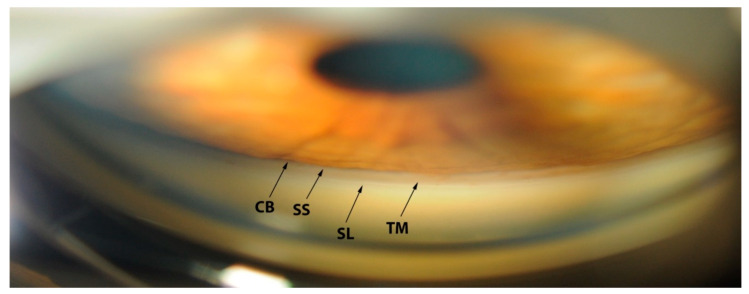
Gonioscopic image of the anterior chamber angle. SL: Schwalbe’s line; TM: Trabecular meshwork; SS: Scleral spur, CB: Ciliary body band.

Gonioscopy has a fundamental role in eye examination, being the clinical reference standard for ACA evaluation [18]. It enables the visualization of angle structures, through a lens or a prism. The angle structures seen with gonioscopy from posterior to anterior are: the iris root, the ciliary body band (CB), the scleral spur (SS), the pigmented and non-pigmented TM and the Schwalbe’s line (SL) [3] (Figure 2).

Direct observation of the ACA structures is not possible due to total internal reflection: light from the ACA strikes the tear–air interface at an angle larger than the critical angle, and thus it is reflected back into the eye. Therefore, from the instrumental point of view there are two potential approaches to perform gonioscopy, both based on an index matching-fluid:

- Direct, in which light from the anterior chamber passes through the cornea and through a contact gonioscopy lens, allowing direct view of the iridocorneal angle.
- Indirect, the gold standard technique, in which light from the anterior chamber is reflected in a mirror allowing an inverted view of the angle [19].

#### Gonioscopic Irido-Corneal Angle Anatomy

The term “iris insertion” is generally referred to the anterior face of the ciliary body, although the site of the insertion may vary. The CB represents the longitudinal fibers of the ciliary muscles. Its width is related to the ACD and may be wide in myopia and phakic eyes or not visible in hyperopia and in case of anterior iris insertion.

The SS is a short extension of the sclera localized between the pigmented TM and the CB. It is noted by its white color during gonioscopy, and is an important landmark for several imaging techniques, such as UBM and AS-OCT [20]. The SC drains the aqueous humor after passing through the TM and is only visible when there is blood inside. The TM is posterior to the SL and extends until the SS, and is an important landmark for diagnostic, incisional and laser surgery purposes. The TM is traditionally divided into an anterior and a posterior portion. The anterior portion lies between the SL and the anterior edge of the SC, while the posterior portion lies between the anterior edge of the SC and the SS. The posterior TM is generally pigmented and is the functional part of the anatomical structure, draining aqueous humor from the anterior chamber towards the SC [21]. The amount of pigmentation at this level is extremely variable and may differ even from quadrant to quadrant in the same person.

The SL, the most anterior structure seen by gonioscopy, is a condensation of collagen tissue between the corneal endothelium and the TM. A pigmented SL may be misinterpreted as TM, particularly in the case of a convex iris profile. Sometimes, a posterior embryotoxon may be seen at the level of the SL, appearing as a ring protruding inside the anterior chamber [22,23]. The presence of a posterior embryotoxon should prompt for careful examination, to identify anomalies related to glaucoma.

Gonioscopy is a subjective technique, has a degree of invasiveness, requiring topical anesthesia, and may be bothersome for the patient. It is a time-consuming examination, requiring experienced operators and a long learning curve. Finally, being a contact technique, it should be avoided in the presence of infectious disorders or a damaged corneal epithelium. Despite all these limitations, gonioscopy is the current gold standard technique for angle assessment, providing a detailed 360° view of the ACA, from the SL to the CB [18,19]. It is worth noting that only dynamic gonioscopy allows for differentiating between permanent and non-permanent appositional angle closure. Indeed, indentation allows evaluating the presence and the extension of peripheral anterior synechiae (PAS), distinguishing between synechial and non-synechial angle closure [24]. This is a key advantage of dynamic gonioscopy in the management of the angle closure spectrum disease.

Several gonioscopic grading systems have been proposed, with the goal of classifying the ACA. The Shaffer’s and the Scheie’s grading systems evaluate the degree of angle opening, while the Spaeth’s system also takes iris insertion and iris configuration into account. Shaffer’s classification model is the most widespread in clinical practice, and differentiates among 5 grades of angle opening (0–4), 0 and 4, indicating irido-corneal contact and an identifiable CB, respectively [19]. Beyond these classification systems, clinical guidelines for the management of angle closure prevalently rely on the extent of irido-trabecular contact (ITC) and the presence of PAS at gonioscopy, rather than on angle grading [24]. This would allow a translation of the ACA assessment across clinicians, aiming at a common management of the angle closure spectrum disease.

It has been demonstrated that the anatomical continuum between the closed and the open angle is hardly represented by any ordinal grading system, and the correlation between ordinal grades and quantitative parameters is probably nonlinear. In 2020 Phu et al. correlated ordinal gonioscopic angle grades with quantitative angle parameters, acquired with AS-OCT [25]. The authors took the wide distribution of AS-OCT parameters into account and identified three meaningful grades of angle opening, that may drive the clinical practice. The “closed” angle combines the gonioscopic grades 0 and 1, the “borderline” angle matches the gonioscopic grade 2, and the “open” angle combines the gonioscopic grades 3 and 4. According to this model, the transition from the gonioscopic grade 1 to the higher grades may be considered a break point in differentiating between closed and open angles.

In recent years, automated gonioscopy techniques have been introduced. In a study by Teixeira et al., manual dynamic gonioscopy was compared to automatic gonioscopy performed with the NGS-I automated gonioscope (Nidek Co., Ltd., Tokyo, Japan), with the goal of assessing the agreement between the two techniques. Angle closure was detected in 23.4% and in 4.3% of the 88 eyes analyzed, by manual and automated gonioscopy, respectively. Since the agreement between the two techniques was poor, the authors concluded that automated gonioscopy needs further improvements for routine clinical use [3].

### 2.2. Ultrasound Biomicroscopy (UBM)

High-frequency ultrasound biomicroscopy, first introduced by Pavlin and Foster in 1989, is a non-invasive technique providing detailed two-dimensional gray scale images of the anterior segment structures in vivo [26]. A voltage transient applied to a piezolelectric transducer generates a focused high-frequency ultrasound wave. Echoes are produced when the wave encounters acoustic impedance discontinuities. The reflections are converted back into voltages by the transducer, amplified by the system electronics and used to produce B-scan images by mechanical scanning of the transducer [27]. A computer system acquires the reflected sound waves, providing high resolution scan images with an axial resolution of 25 μm, lateral resolution of 50 μm and a depth of tissue penetration of a few millimeters (3–5 mm) [26,28]. Higher frequencies are usually associated to a higher image resolution with the disadvantage of a poorer penetration [29,30,31]. Currently, most commercial UBM systems uses frequencies up to 50 to 100 MHz [30]. The 50 MHz probe is able to penetrate 4 mm, has a resolution of 40 μm and is thought to balance the best depth and resolution [30,32]. The procedure is alike the conventional B-scan ultrasonography, but needs an immersion scleral shell or a disposable ClearScan water balloon on the probe tip [32]. The angle structures such as iris, ciliary body and SS can easily be identified by UBM, with SS being a landmark for various quantitative measurement parameters [33]. AOD, ARA, TIA and TCPD are the most commonly used parameters for the assessment of the ACA [16,34] (Table 1)

The agreement between UBM and gonioscopy has been evaluated in several studies [35,36]. Radhakrishnan et al. assessed the accuracy of UBM and of an AS-OCT prototype (4000 axial scans/s) for the detection of narrow angles. The ACA parameters measured by both UBM and AS-OCT had similar values, sensitivity, specificity, and reproducibility. Both techniques showed excellent performance in identifying eyes with narrow angles, with areas under the receiver operating characteristic curves (AUC) in the 0.96–0.98 range, for all the parameters analyzed [37]. Despite these data, AS-OCT has undoubtedly significant advantages over UBM, allowing for faster acquisition and better resolution of the images. Moreover, positional variation, failure to control accommodation and room illumination are all factors that may influence UBM image acquisition. To overcome, at least partially, these limitations, it has been suggested to standardize patient’s gaze direction by placing some markers inside the room [38], and to control room illumination before starting UBM acquisition. However, other confounding factors are not amendable, such as the subjective influence by the operator [33].

Barkana et al. compared UBM with gonioscopy for the detection of ITC, under normal room illumination and subsequently in dark-room lighting. ITC was found in 16 out of the 18 superior quadrants (89%), with appositional closure during dark-room gonioscopy, but in only 6 (33%) of the 18 superior quadrants evaluated under normal room illumination. These results support the recommendation that both imaging techniques should be performed under dark-room illumination in order to avoid misdiagnosis of ITC [35]. To evaluate the association between visibility of the TM at gonioscopy and appositional contact at UBM, a population-based study was conducted in a cohort of Chinese subjects, with gonioscopic primary angle-closure suspicion (non-visible posterior TM in ≥2 quadrants at static gonioscopy). In this study, a larger ITC was detected by UBM than gonioscopy [36].

**Figure 3:**
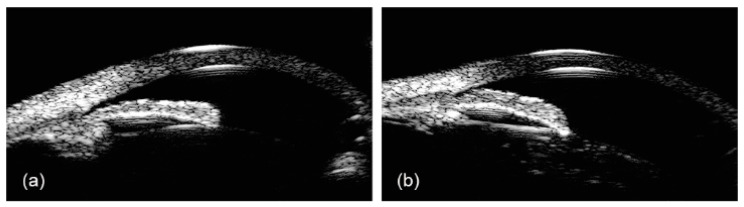
Ultrasound biomicroscopy images of narrow angle (**a**) and plateau iris configuration (**b**).

Opposite to AS-OCT, that uses electromagnetic waves (light), the sound waves of UBM penetrate the pigmented epithelium, allowing an evaluation of the structures behind the iris pigment epithelium, such as the lens zonules, the ciliary body and the posterior chamber [39,40,41]. UBM also allows examining the in-vivo interaction among structures of the anterior segment, to better understand the mechanisms of angle closure and the causes of secondary glaucoma, such as lens subluxation, iridociliary cysts, tumors, and plateau iris configuration. In this latest condition, UBM is extremely useful to identify the ciliary body and characterize its position, being the gold standard technique in comparison to AS-OCT [42] (Figure 3).

In pigment dispersion syndrome, UBM has been used to show iris concavity and the changes of anterior lens surface during accommodation [43]. UBM also allows a better visualization of flare and cells into the anterior chamber, defining the severity and the extension of intraocular inflammation associated with glaucoma [44]. Moreover, Wang et al. used UBM for ciliary body measurements, in eyes developing malignant glaucoma after trabeculectomy, and discovered that patients had on average thinner and anteriorly rotated ciliary body in both eyes, a potential predisposing factor to the disease [45].

The effect of age and body position on angle width, measured by means of UBM, has been investigated in two cohorts of young (18–30 years) and elderly subjects (>45 years) [46]. In the younger cohort, no difference in angle width was found, as related with measurement location (superior vs. inferior) and body position (sitting/supine). Instead, an interaction between body position and measurement location was found in the elderly cohort, with deeper angle inferiorly and superiorly, respectively, in the sitting and supine position.

Previous studies have reported good to excellent intra-observer reliability using UBM, but poor inter-observer reliability in measuring the ACA and iris dimensions (AOD, TIA, ARA, iris thickness (IT)) [38,47,48,49]. These results may be explained by considering the subjective definition of the anatomical landmarks (e.g., the SS), against which the data from quantitative measurements of the angle are collected. Moreover, inconsistencies in the alignment of the probe may be a source of variability, especially among different operators [38].

Automated assessment of the angle structures may be useful to screen out subjects with closed or narrow angles, and then refer them to experienced specialists for further examination. Recently, some studies have reported encouraging results in the development of automated image assessment software [50,51]. Li et al. proposed a learning-based method to fully automate the measurement of TIA with UBM, without manual positioning of the SS. This automated algorithm demonstrated high accuracy and good consistency with manual assessment (ICC of 0.93–0.99, CV of 6.4–8.8%), suggesting a role in future clinical practice [52]. However, no data were made available regarding the ability of this algorithm to measure TIA in eyes where anatomical landmarks may be difficult to set, such as in very narrow or closed angles.

### 2.3. Gonio-Photographic Systems (GPS)

Several GPSs have been developed in recent years. These include two commercially available devices, the EyeCam (Clarity Medical Systems, Pleasanton CA, USA) and the NGS-1 automatic gonioscope (NIDEK Co., Gamagori, Japan), and two prototypes, the GonioPEN and the Axicon lens- assisted gonioscope. Most of them require several images to select the best one, whereas NGS-1 performs this operation automatically [17].

The EyeCam is a portable hand-held device taking images of the ACA. It was originally designed to acquire wide-field images of the retina and its use was then extended to the ACA. As with slit-lamp gonioscopy, it requires contact with the eye through a 120° or a 130° wide-field lens. The hardware consists of a hand-held digital video camera connected by an optical fiber bundle to a light-emitting control unit and a computer assembly [53]. In contrast to gonioscopy, the patient lies supine, under dark-room illumination, for the whole duration of the examination. A coupling gel is placed on the probe, minimizing discomfort and compression artefacts. The patient is asked to look in the direction of the studied angle, whereas the probe is positioned at the opposite limbus [31]. The operator checks the amount of light and the focus, and images and videos are automatically captured and saved into a computer hard drive [53]. EyeCam is therefore a new objective method documenting angle anatomy, similarly to gonioscopy [31]. The rate of poor quality images was found to be absent or minimal in previous studies, as EyeCam achieved clear angle images in 98 to 100% of the subjects [53,54]. A good agreement between EyeCam and gonioscopy has been shown in several studies [51,53,55]. Perera et al., in 2010, compared EyeCam with gonioscopy for the detection of closed angles, defined as angles where the TM could not be seen in ≥2 quadrants. The agreement between gonioscopy and EyeCam, based on AC1 statistics, was 0.73, 0.75, 0,76 and 0.72, for the superior, inferior, nasal and temporal quadrants, respectively. Less eyes were diagnosed as closed angles with gonioscopy (21/152, 13.8%) than with EyeCam (41/152, 27.0%) (*p* < 0.001, McNemar test). This discrepancy could be related to differences in illumination, as well as to difficulties to explore angle structures in eyes with a convex iris profile. Sensitivity and specificity of EyeCam were 76.2% and 80.9%, respectively, with an AUC of 0.79 for the detection of angle closure [53].

EyeCam diagnostic ability for the detection of angle closure has been compared with slit-lamp gonio-photography, assuming gonioscopy as the reference standard. In a cohort of Chinese subjects, angle closure (≥2 closed quadrants of angle circumference) was identified in 38/85 eyes (45%) using gonioscopy, 40/85 eyes (47%) using EyeCam and 40/85 eyes (47%) using gonio-photography (*p* = 0.069 for both the comparisons, McNewman test). In this study, the authors demonstrated better agreement between gonioscopy and EyeCam (k = 0.86; 95% CI: 0.75–0-97) than previously found [53]. Moreover, in a study on 98 phakic subjects, EyeCam showed better diagnostic performances for the detection of angle closure (≥2 gonioscopically closed quadrants of angle circumference) than Visante AS-OCT (Carl Zeiss Meditec, Inc.; Dublin CA, USA), with AUC values of 0.978 (95% CI: 0.93–1.0) and 0.847 (95% CI: 0.76–0.92), respectively (*p* < 0.001). In this study, EyeCam achieved a specificity and a sensitivity of 95%, significantly higher than AS-OCT (92% and 65%, respectively) [54].

Baskaran et al. proposed a new algorithm to provide an automatic classification of EyeCam angle images. In eyes with a gonioscopic diagnosis of angle closure (posterior TM not visible in ≥2 quadrants of angle circumference), the manual classification and the new automated grading algorithm showed similar diagnostic performance (AUC 0.974 and 0.954, respectively; *p* = 0.31). Agreement between gonioscopy and both the manual and the automated image classification was good, when taking into account the whole angle circumference (k = 0.88, 95% CI: 0.81–0.96 and k = 0.74, 95% CI: 0.63–0.85), but poor when individual quadrants were considered. Misclassification of open and closed angles was associated with heavy or light pigmentation, and partial angle closure [51].

To quantify angle anatomic variations, Xu et al. compared the image grading achieved with EyeCam, gonioscopy and Swept-Source Optical Coherence Tomography (SS-OCT) in a population-based cohort of Chinese Americans. As a result, EyeCam images significantly under-represented inter-quadrant differences in ACA configuration, when compared to SS-OCT. Moreover, significant disagreement was found between the gonioscopic and the EyeCam angle grading, suggesting a reconsideration of the current methods for angle closure diagnosis [56]. These results may be due to the decrease of structure visibility in narrow angles, as the direct visual assessment of angle anatomy may not accurately reflect its depth, as with AS-OCT [56]. On the other hand, the supine position, required for image acquisition, may influence EyeCam results. Indeed, previous studies with UBM have demonstrated position-related changes of the ACA configuration, when shifting from sitting to supine [46].

The inter- and intra-observer reliability of EyeCam for the detection of angle closure was reported to be moderate to excellent in a large population-based study (k = 0.82 and k = 0.87, respectively) [57].

In conclusion, EyeCam is an easy and objective method providing a good visualization of the entire circumference of the ACA, similarly to gonioscopy. However, its inability to provide dynamic indentation makes the interpretation of angle images difficult, especially in the case of narrow angles, convex iris configuration or light TM pigmentation. Despite being unable to provide quantitative measurements of the ACA, EyeCam may add valid information for the follow-up evaluation of glaucoma patients [31]. A potential advantage of this realistic assessment technique may be the ability to quantify ACA pigmentation, by means of image analysis software. These data are helpful especially during the follow-up of pigment dispersion syndrome and pigment dispersion glaucoma, generally characterized by a late burn-out phase [58]. Indeed, in the late burn-out phase, pigment dispersion may be absent, and intraocular pressure normalized. For this reason, pigmentary glaucoma may be misdiagnosed as primary open-angle glaucoma, or normal tension glaucoma, with diagnostic and therapeutic flaws.

The automated NGS-1 gonioscope acquires full circumferential 360° gonioscopic images of the ACA. NGS-1 hardware includes a rotating reflecting 16-faceted optical contact prism, an illumination provided by white LEDs and a high-resolution color camera. Each of the 16 prism mirrored facets projects a white light onto a 22.5° portion of the ACA. The camera takes 17 pictures at varying focal depths, from each of the 16 gonioprism facets. Examination of the eye, including manual focusing time, takes about 1 min, thus requiring good fixation and reasonable patient cooperation. A previous study reported that 28 of the 336 sections (8.33%) acquired with NGS-1 had to be excluded, due to poor image quality after manual selection [59], whereas low quality images have been reported up to 22.5% of cases [3]. Angle closure was detected in 4.3% and in 23.4% of the eyes with NGS-1 automated gonioscopy and dynamic gonioscopy, respectively, in a study by Teixeira et al. These results demonstrate that the instrument may have low sensitivity in comparison with gonioscopy [3]. The inter-rater reliability of standard and automated gonioscopy for angle closure detection is modest, with a Fleiss’kappa of 0.17 (95% confidence interval: 0.035–0.238) [3].

The GonioPEN is a gonioscope connected to a PC through a USB port, combining high-resolution color camera and LED illumination. The camera captures images of the eye from 4 different perspectives. The imaging probe is connected to a slit-lamp and placed near the limbus to examine the opposite ACA. Using a coupling gel, the camera is able to visualize angle structures in a way similar to direct gonioscopy [60]. The use of an axicon lens may improve the gonioscopic image resolution. Improvement in the resolution is characterized in terms of Huygens point spread function, with distinct separation of the 3 μm point sources. However, even though improvements may be noted with the use of an axicon lens, other issues such as lighting control, magnification adjustments and expertise requirement remain unsolved [61].

## 3. Non-Contact Techniques

### 3.1. Limbal Anterior Chamber Depth Measurement (LACDM)

**Figure 4:**
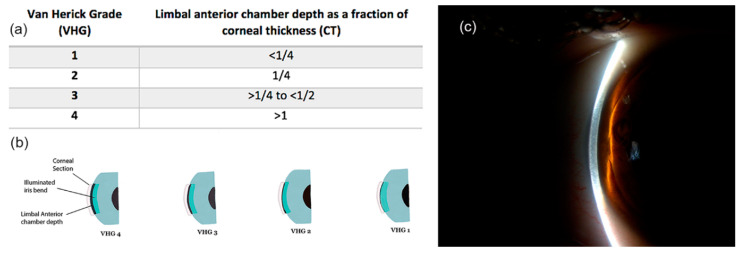
Limbal anterior chamber depth measurement using traditional Van Herick grading (VHG) system. (**a**,**b**) Table and illustration of the traditional 4-point Van Herick grading scheme; (**c**) Slit-lamp image of LACDM.

Slit-lamp evaluation of the ACD with Van Herick technique is a non-contact approach for the estimation of angle width. The illumination column of the slit-lamp is offset from the central axis by 60° to the temporal side of the microscope. A narrow light beam of light is directed to the ocular surface at limbus, and an ACD measurement is carried out by comparing the space between the endothelium and the anterior iris surface with the peripheral corneal thickness [33]. The traditional van Herick grading system provides a 4-point grading scheme, in which limbal ACD is graded ≤25% (VHG 1), 25% (VHG 2), >25% and ≤50% (VHG 3), and >100% of the corneal thickness (VHG 4) [62] (Figure 4).

The Van Herick technique has shown variable levels of accuracy for the detection of angle closure [63,64,65]. Thomas et al. showed that the traditional 25% cut-off (grade ≤2) has a specificity of 89.3%, but sensitivity is only 61.9% [63]. In another study, a sensitivity of 56% and a specificity of 95% were found using the same Van Herick cut-off, suggesting that this technique may be a poor predictor of angle closure [64]. In a Japanese population-based study, 86.3% and 64.2% of the eyes classified as Van Herick grade 1 and Van Herick grade 2, respectively, had an angle narrow as or narrower than Shaffer grade 2 [65].

In the year 2000, Foster et al. proposed a modified Van Herick grading system, trying to increase the diagnostic accuracy of the methodology. The original grade 1 was split into three sub-grades (i.e., 0%, 5% and 15%), and a new grade was introduced (i.e., 75%), to compensate for the gap between the traditional grades 3 and 4. In a population-based study from Mongolia, the authors found that the 15% cut-off (equal to the traditional grade 1) had a sensitivity and specificity of 84% and 86%, respectively, for the detection of occludable angles [66]. However, Baskaran et al., using the same 15% cut-off, found a sensitivity of 60.4%, in front of a 100% specificity. In this same study, the 25% cut-off achieved a sensitivity of 84.9% and a specificity of 89.6% [67].

Recently, Sihota et al. proposed a “Van Herick Plus” classification system, i.e., a modified grading scheme involving a short vertical slit beam, and reported a good correlation between this method and AS-OCT measurements, for the identification of occludable angles [68].

Good inter-observer reliability of the Van Herick technique has been previously reported [63,66]. However, although good reliability has been found for angles classified as grade 1 and 4, the same was not true for angles classified as grade 2 and 3, likely requiring further examinations to increase reliability [69]. These results are somehow not surprising. Indeed, previous studies have demonstrated a poor correlation between gonioscopic angle grading and other quantitative measurements, when evaluating angles that are at the edges of the ordinal gonioscopic scale, i.e., closed/narrow angles and wide-open angles [25,70]. As a consequence, while differentiating between very narrow and wide-open angles may be obvious for the clinician, and consequently highly repeatable, classification of the anatomical continuum between these two extremities into an ordinal gonioscopic scale may be difficult, if not impossible [70,71].

Limbal ACD assessment using the Van Herick technique is an easy, rapid and non-contact technique, allowing for the identification of gonioscopically closed angles [33]. It does not require a long learning curve and may be useful also for non-glaucoma specialists, being accessible to everyone with a slit lamp. A recent Cochrane review underlined the importance of this technique for the identification of occludable angles in high-risk populations [72]. Interestingly, Van Herick assessment showed similar diagnostic performance in comparison with more advanced and invasive technologies, such as AS-OCT, Scheimpflug camera and SPAC. For this reason, Van Herick technique may be a valuable tool, especially in the case of limited access to the most recent technologies. On the other hand, the Van Herick technique remains a non-standardized examination, with regards to the illumination column, the slit height and the exact placement of the slit at the peripheral cornea. Moreover, it gives no information about the shape of the peripheral iris, the appearance of the angle structures, the presence of PAS and their extension [68]. Finally, it is not doable in eyes with corneal opacities at the temporal limbus [73,74].

### 3.2. Scanning Peripheral Anterior Chamber Depth Analyzer (SPAC)

The Scanning Peripheral Anterior Chamber (SPAC) depth analyzer is an optical system developed to provide an objective method for the detection of eyes at risk of angle closure [75]. SPAC is a non-contact instrument, made of a slit-lamp microscope orientated at 60° to the optical axis of the eye. Moving perpendicularly to the eye axis, the instrument is able to acquire a series of corneal and iris images, then processed to calculate ACD, and to give both quantitative and categorical classifying grades [67].

A large clinical-based Japanese study revealed that SPAC has high accuracy in the detection of eyes at risk of angle closure glaucoma, with a 97.6% sensitivity and a 83.5% specificity [75]. However, other studies found that both the categorical and the numerical SPAC grading systems have only a moderate agreement with the Van Herick technique, when evaluating peripheral ACD [67]. Taking gonioscopy as a reference, the AUC for the SPAC categorical grade S (suspect angle closure) or P (potential angle closure) was 0.790, with an 84.9% sensitivity and a 73.1% specificity. When the reference was a modified version of the Van Herick technique (cut-off of peripheral ACD ≤25%), SPAC achieved an AUC of 0.872, with an 84.9% sensitivity and an 89.6% specificity.

Despite being advocated as a quick and potentially easy screening tool for the detection of angle closure glaucoma, SPAC identifies more narrow angles (63/120) than gonioscopy (53/120), and Van Herick (52/120) [67]. Lavanya et al. evaluated SPAC as a screening tool for the identification of gonioscopic narrow angles (i.e., posterior pigmented TM visible in ≤2 quadrants of angle circumference), in a population of 2052 subjects. Using grade 5 as a cut-off, the AUC was 0.83 (95% CI: 0.82–0.85), with a sensitivity of 90% and a specificity of 76%. The authors concluded that a so-low specificity may limit the use of this device as a screening tool for narrow angles [76].

### 3.3. Scheimpflug Photography (SP)

**Figure 5:**
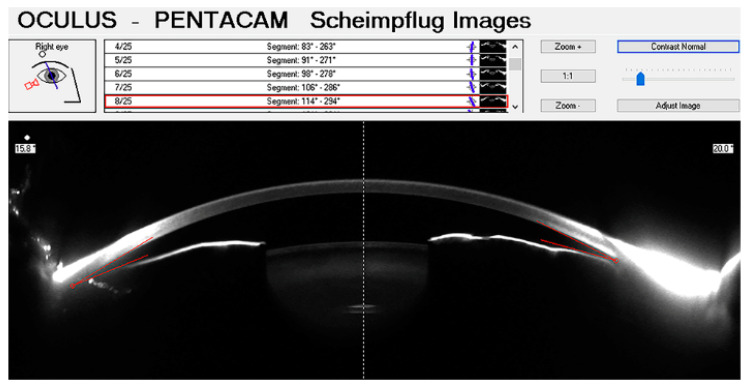
Anterior chamber image acquired with Pentacam, and quantitative measurements provided by the built-in software.

The Scheimpflug principle is based on a geometrical optical concept, involving a non-parallel orientation of the lens and image planes, to correct for perspective distortion [77]. Applied to ophthalmology, it allows the acquisition of anterior segment images, with a depth of focus ranging from the anterior cornea to the posterior lens surface [33,78] (Figure 5).

Pentacam is a non-contact instrument that uses the Scheimpflug principle to acquire anterior segment images. It includes a rotating Scheimpflug camera that allows for 3D-analysis of the anterior chamber and for measurements of corneal pachymetry, corneal diameters, lens position, curvature radius, ACV and ACD [19]. The acquisition procedure is fast and allows performing 12 to 50 single cross-section photographs from 0° to 360° in approximately 2 s [79,80]. After the acquisition, the device detects and processes up to 25,000 height values generating a 3D virtual model of the anterior segment of the eye [79].

Pentacam is a valid tool for the evaluation of angle closure, offering a quantitative analysis of ACD and ACV [19]. It has been used to directly measure the effect of pilocarpine on ACD and ACV in eyes with narrow angle, and to analyze the dynamics of the anterior chamber after peripheral iridotomy [19]. However, Pentacam does not allow for direct visualization of the angle recession, due to the inability of light to deeply penetrate the tissues. Moreover, since the iris plane is defined using a straight line, the angle width measurement may result inaccurate [18]. Although previous studies have shown good intra-observer and intra-session repeatability of ACA measurements with Pentacam [81,82,83], the effect of confounding factors, such as lighting condition and accommodation, and both the inter-observer and the inter-session repeatability have been poorly explored.

Several studies aimed at assessing whether SP may be effective as conventional techniques in ACA evaluation. Grewal et al. reported an AUC of 0.93 (95% CI: 0.90–0.96), for the detection of narrow angles with Pentacam [84]. In a study by Kurita et al., Pentacam was compared with UBM and gonioscopy for the diagnosis of eyes with primary angle closure. UBM measurements of the ACA had the highest correlation with the Shaffer grading system; in distinction, Pentacam turned out to be not so effective in the evaluation of angles with a Shaffer grade of 2 or less, likely due to the limited angle visualization. Nevertheless, Pentacam was found to play a role in the screening of eyes with primary angle closure or primary angle closure suspicion, based on ACD and ACV measurements [85,86].

Despite these data, the evaluation of the ACA with SP has some limitations when compared with AS-OCT, due to its inability to directly visualize the SS [19,86]. For this reason, Pentacam parameters show poor correlation with gonioscopy and UBM data in closed angles [87,88].

Other studies have been conducted with different imaging techniques relying on the Scheimpflug principle. Ruiz-Belda et al. evaluated the intra-session repeatability of ACA measurements in healthy eyes, using the Sirius Scheimpflug photography-based system (glaucoma analysis mode). Measurements were taken at the nasal and at the temporal meridians in 43 eyes, concluding that the Sirius system provides a reliable irido-corneal angle evaluation, and can be considered a valid non-invasive technique for the detection of occludable angles [89].

A major issue with SP and other non-contact imaging techniques (e.g., AS-OCT), is the lack of normative databases, against which the acquired data may be compared. Other than decreasing diagnostic accuracy [71], this may be perceived as a strong limitation, especially by the general clinician in search of clear advice in every-day clinical practice. Building a normative database for these instruments may be challenging. Indeed, since ACA parameters are influenced by age, gender, ethnicity, refractive errors, and other anthropomorphic characteristics, specific patterns of changes related to these factors should be acknowledged, in order to derive normative models, useful in the diagnosis of anterior chamber pathological changes [71].

### 3.4. Anterior Segment Optical Coherence Tomography (AS-OCT)

AS-OCT is a non-invasive technique acquiring high-resolution images of the ACA, allowing for both quantitative and qualitative analyses [16,90] (Table 1).

Optical coherence tomography uses low-coherence interferometry to provide cross-sectional images of the ocular tissues. The interferometer can be based on the time (TD-OCT), the spectral (SD-OCT) or the swept source (SS-OCT) domain [91]. Two- or three-dimension tomographic images are acquired by measuring the interference between the light scattering from tissue structures and a reference optical signal. Light emitted by a low-coherence source is split into the two arms of an interferometer, where illumination properties such as depth of focus and intensity distribution of the beam are defined. Light is backscattered by the interface between tissues with different refractive indexes, and returns through the reference and the sample arms, recombining at the beam splitter on their way to the detector. Interferences only occur when the pathway between the two beams is within the coherence length of the light source [92]. Whereas in time-domain configuration (TD-OCT) light echoes are detected sequentially, spectral domain OCT (SD-OCT) collects modulations in the source spectrum with all the spectral components captured simultaneously, so that a higher scan rate is achievable (up to 100,000 A-Scans per second), when compared to TD-OCT (200-2000 A-Scans per second) [92,93].

Swept source approach is the most recent implementation of OCT. In SS-OCT, the light source is a fast-tunable laser (swept source) emitting narrowband light, with variable wavelength at a high rate. This way, the sample tissue is sequentially exposed to several wavelengths. The interference patterns are detected one by one and stored in a buffer. After a complete sweep of the tunable light source, the interferogram recorded over time is processed with an inverse Fourier transformation, to achieve an A-scan, similarly to SD-OCT. The difference between SS- and SD-OCT lies in the way the different wavelengths are generated and recorded: whereas in SD-OCT all the light components are generated and detected at the same time, in SS-OCT a spectral range of wavelengths is covered by the light source, allowing for a greater axial range, and a faster acquisition, with more than 400,000 A-scans/s.

Nolan et al. evaluated the ability of a time-domain AS-OCT prototype (Carl Zeiss Meditec, Inc., Dublin, CA, USA) to detect primary angle closure in Asian people. Angle closure, defined as the contact between peripheral iris and the angle wall anteriorly to the SS in ≥1 quadrant, was detected in 228 of 342 eyes (66.7%) by AS-OCT, and in 152 of 342 eyes (44.4%) by gonioscopy. AS-OCT resulted as highly sensitive for the diagnosis of angle closure. The disagreement between AS-OCT and gonioscopy was explained by considering that, unlike gonioscopy, AS-OCT uses infrared light and does not cause inadvertent indentation, that may open the ACA [94]. A community-based study on 423 subjects compared the performance of Visante AS-OCT and gonioscopy in diagnosing angle closure. The angle was found to be closed (posterior TM not visible at gonioscopy) in at least 1 quadrant of the 59% and 33% tested eyes, with AS-OCT and gonioscopy, respectively (*p* < 0.001). The tendency of AS-OCT to identify angle closure more often than gonioscopy could be explained taking into account the variations of the iris profile, and the presence of small areas of ITC, not graded as closed at gonioscopy [95]. Interestingly, a previous study performed with Visante AS-OCT demonstrated that ACA parameters may vary by race [96]. In this study, Vietnamese (*n* = 126) subjects had smaller values for all the ACA parameters, in comparison to Chinese (*n* = 124) and Caucasian subjects (*n* = 112) (*p* < 0.01). The only exception was the lens vault (LV) value. When these authors considered only eyes with open angles, again Vietnamese people showed smaller angles and anterior chamber dimensions (*p* < 0.01), as well as thicker iris (*p* < 0.01), in comparison to Caucasian people. These results provide evidence to the difficulties associated with the development of normative databases for ACA measurements, taking into account the variability of ACA parameters by age, race, and other biometric factors.

To investigate whether AS-OCT could predict incident gonioscopic angle closure in a 4-year follow-up, Baskaran et al. enrolled a cohort of 342 subjects with gonioscopically open angle but ITC detected by Visante OCT. As a result, subjects with more quadrants of AS-OCT angle closure at baseline had a greater chance of developing gonioscopic angle closure after 4 years of follow-up (*p* < 0.0001) [97]. To explore the baseline AS-OCT parameters associated with incident angle closure, 342 open-angle subjects were recruited in a Chinese community-based study. Small values of AOD at 750 µm, defined as the perpendicular distance between a point 750 µm anterior to the SS and the opposing iris, and higher values of LV, were associated with angle closure development, during the 4-year study follow-up (odds ratio: 1.29 (95% CI: 1.07–1.57) and 3.27 (95% CI: 1.87–5.69), for a 0.1 mm increase of AOD750 and LV, respectively) [98].

Chang et al. evaluated the diagnostic ability of a sequential testing methodology with SPAC and Visante AS-OCT. In their study, a 70.3% sensitivity and a 94.3% specificity for the detection of narrow angles were reported, using the sequential SPAC and AS-OCT testing methodology. In distinction, AS-OCT alone achieved a sensitivity of only 52.1%, suggesting that sequential tests may be useful in a screening setting [99].

**Figure 6:**
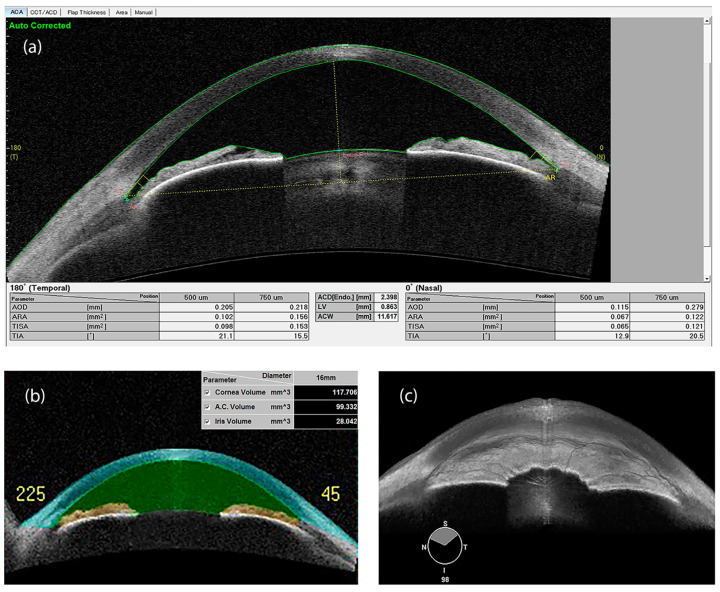
Swept source-OCT image of the anterior chamber, with scleral spur identification and quantitative parameters, in an eye affected with narrow angle and keratoconus (**a**). Anterior chamber volume (**b**) and 3D rendering of a narrow irido-corneal angle (**c**) (swept-source OCT). ACD: Anterior chamber depth; ACW: Anterior Chamber Width; AOD: Angle opening distance; ARA: Angle recess area; LV: Lens vault; TIA: Trabecular-iris angle; TISA: Trabecular-iris space area.

While short axial length and shallow ACD are well-known risk factors for the development of angle closure, AS-OCT has contributed to highlight other parameters that may be associated with angle closure, namely reduced values of the anterior chamber area (ACA) and ACV [100], greater LV [101], larger IT and iris curvature (IC), and abnormal iris configuration [102] (Figure 6). Among these parameters, ACA, ACV and LV have been reported as the most relevant determinants of angle width, in a large population-based study [103]. Moreover, Cheung et al. showed that iris bowing is a biometric parameter significantly associated with angle width, independently of ACD. In this perspective, evaluating dynamic changes of the iris configuration may offer a new way towards the evaluation of angle-closure risk [104].

SS-OCT CASIA SS-1000 (Tomey Corporation, Nagoya, Japan) was the first instrument allowing a circumferential assessment of the ACA, with moderate to good diagnostic performance in comparison to gonioscopy [17]. In a few seconds (2.4 s), SS-OCT acquires 128 meridional scans (256 images) of the ACA circumference, granting a 3-dimensional analysis of the entire structure [105]. SS-OCT has been found to be accurate and reproducible in quantifying the amount of circumferential ITC, as well as the amount of PAS. Indeed, Lai et al. reported a good agreement of SS-OCT and gonioscopy in the assessment of PAS (k = 0.79; 95% CI: 0.67–0.91) [106].

The diagnostic performance of SS-OCT in the detection of gonioscopic angle closure (i.e., iris contact with any portion of the angle wall, anteriorly to the SS), has been investigated in a large community-based study [107]. The AUC of SS-OCT was 0.84 (95% CI: 0.81–0.88), indicating a moderate diagnostic performance, similar to Visante AS-OCT (AUC: 0.76; 95% CI: 0.74–0.78) [76,107]. In a study by Melese et al., 9 out of the 24 SS-OCT tested parameters had no misclassification of narrow angles (sensitivity = 1.0), with a specificity >0.79 [108]. In this study, narrow angles were gonioscopically defined as angles with no visible posterior TM, while open angles were defined as angles that are open, up to the SS and beyond. Agreement between gonioscopy and SS-OCT was investigated in a study by Rigi et al., on 86 eyes with open or narrow angles [109]. Based on the Spaeth gonioscopic grading system, “A” and “B” angles were classified as narrow, “C”, “D”, and “E” as open. Angle evaluation was performed exploring the superior quadrant, by two masked examiners, during 2 visits six-month apart. While inter-visit and inter-observer repeatability was moderate to excellent (k: 0.57–0.85), agreement between gonioscopy and SS-OCT was only fair to good (k: 0.34–0.63), with SS-OCT classifying more angles as narrow than gonioscopy.

Using SS-OCT, Tun et al. examined the circumferential reduction of ITC in eyes with synechial primary angle closure glaucoma and cataract, after phacoemulsification with intraocular lens implantation alone or with goniosynechialysis. Authors found that the ITC area was significantly reduced after phacoemulsification with goniosynechialysis, in comparison to phacoemulsification alone (10.2 mm^2^ vs. 4.6 mm^2^, β = 0.54, *p* = 0.03). All the analyses were adjusted for gender, age, PAS extension, intraocular pressure and pupil diameter [110].

In a recent study by Li et al., a model consisting of AOD-750, IT and a new parameter, iris volume (IV), showed great diagnostic ability for the detection of angle closure [111]. All these parameters were measured using SS-OCT. Interestingly, IV and its indirect estimation from 2-dimensional iris cross-sectional area, had been previously identified as a potential risk factor for angle closure, when evaluated as related to pupil dilation [112]. Geometric estimates predict IV to decrease as the pupil enlarges. Consequently, subjects whose IV decreases less with pupil dilation, may be more likely to occlude their TM [112].

A semi-automatic procedure for the evaluation of SS-OCT images has been developed, allowing for the estimation of total ITC, after manual identification of the SS. The software has shown good diagnostic performance for the detection of angle closure (AUC 0.83; 95%CI: 0.76–0.89), i.e., alike manual assessment. As a result, it may help the busy clinician in the interpretation of the acquired SS-OCT images [105].

SS-OCT has been also used to evaluate the effects of aging on the TM of healthy people [113]. Enrolled subjects were divided into three groups, aged 18–40, 41–60 and 61–80, respectively. The TM area, the TM length and the TM interface shadow length did not correlate with age, race, gender, intraocular pressure and gonioscopic grading. Instead, the TM interface area was found to significantly increase with age. As the TM interface shadow may be the result of the reflection of light off the TM, its increase may indicate an augmented density of the TM with age, and consequently, an increased outflow resistance.

AS-OCT measurements of the ACA have shown good intra- and inter-observer reproducibility, independently of the used domain [114,115,116]. However, most if not all these measurements require a landmark, against which the quantitative evaluation is performed. Ideally, this landmark should be easily detectable and minimally influenced by confounding factors, such as light variation, treatment and/or time. Among these factors, especially light variation has shown to greatly influence the ACA measurements [117,118]. Current technological limitations require, in most of the commercially available instruments, manual positioning of the anatomical landmarks, introducing potential measurement errors. It has been demonstrated that the SS, traditionally considered the best landmark for ACA measurements, may be non-detectable in 15–28% of the AS-OCT images, especially in the presence of narrow angles [119,120]. Indeed, inconsistency between operators in identifying the position of the SS accounts for 50% of the TISA variability [121]. These results are likely to be better when using SS-OCT, due to the higher image resolution [122]. To ameliorate the reproducibility of ACA measurements, some authors proposed the SL as a new anatomical landmark. Using SD-OCT (Cirrus, Carl Zeiss Meditec Inc.), a study reported identification of SL and SS in 95% and 85% of angle quadrants, respectively (*p* = 0. 035). SL angle opening distance (SL-AOD) and SL trabecular-iris space area (SL-TISA) measured 500 μm from the SL, were significantly correlated with SS parameters (all *r* values >0.85) and gonioscopic grading (all *r* values >0.69) [123].

It has been demonstrated that the ability to identify the anatomical landmarks may vary with race and angle width. In a study by Crowell et al., masked readers evaluated 2-dimensional SS-OCT images to identify three anatomical landmarks (TM, SC and a band of extra-canalicular limbal lamina (BELL)) in eyes of a mixed-race population [124]. The outer border of the BELL, the TM and the SC were identifiable in the 95%, 73% and 40% of the angles, respectively. However, the outer border of the BELL was more easily detectable in white (97%) than in Asian (82%) subjects (*p* = 0.02), and in eyes with a gonioscopy grade “E” (98%), compared to eyes with a gonioscopy grade “A” (83%) (*p* = 0.02). Similarly, SC was more easily identifiable in open than in narrow angles (43% vs. 27%) (*p* = 0.02).

### 3.5. Deep Learning

Deep learning is an increasingly popular range of techniques in the field of image analysis, focused on specific problems, such as object classification and face detection [125]. In medicine, deep learning algorithms, such as convolutional neural networks, offer fascinating perspectives for the automation of medical image analysis [126]. Recently, artificial intelligence systems have been developed, allowing for the qualitative and quantitative analysis of the anterior chamber structures, by means of deep learning algorithms applied to AS-OCT images [17].

Fu et al. applied a deep learning algorithm to the detection of angle closure on 4135 AS-OCT images. The diagnostic ability of this algorithm was compared to another automated angle closure detection system, based on quantitative features. Both the deep learning algorithm and the quantitative automatic angle closure detection system were evaluated against the clinician’s grading of AS-OCT images. The AUC of the quantitative system was 0.90 (sensitivity 0.79 ± 0.037, specificity 0.87 ± 0.009), whereas the AUC of the deep learning algorithm was 0.96 (sensitivity 0.90 ± 0.02, specificity 0.92 ± 0.008), showing better results in favor of the deep learning technique [127].

In another recent study, Xu et al. developed and tested deep learning classifiers for the detection of gonioscopic primary angle closure. These authors developed 3 competing multi-class convolutional neural network classifiers for the identification of modified Shaffer grades 0, 1, 2, 3, and 4. The best-performing classifier achieved an AUC of 0.93 on the cross-validation dataset. Angle-closure disease was diagnosed with an AUC of 0.96 and 0.95, respectively for the identification of angle closure in 2 and 3 quadrants of angle circumference. These results are encouraging, as they demonstrate the ability of deep learning classifiers to perform a sort of “automated gonioscopy” on AS-OCT images, with similar accuracy of an experienced and highly trained ophthalmologist (~4000 gonioscopic examinations) [128].

Other deep learning algorithms are under development, aiming at automatically identifying angle anatomical landmarks in AS-OCT scans (e.g., the SS) [129,130,131]. These algorithms may guarantee an objective evaluation of angle quantitative parameters, helping in the diagnosis of angle closure. However, their diagnostic ability has still to be tested on large mixed-race populations and for very narrow angles, where even the manual identification of anatomical landmarks may be difficult [132].

## 4. Conclusions and Future Directions

The aim of this review is to stress the importance of ACA investigation in any comprehensive ophthalmological visit, as well as to show the plethora of currently available diagnostic techniques. Nowadays, a wide range of instruments allows the ophthalmologists exploring the ACA configuration, helping in the detection of narrow angles and in the diagnosis of angle closure (Table 2). Among traditional contact techniques, gonioscopy has been consistently considered for a long time the gold standard of ACA evaluation, although it is a time-consuming examination, requiring a long learning curve and good ocular surface conditions. Similarly, the UBM examination, even more suitable to identify anterior angle structures, requires contact with the eye and a well-trained operator to be properly carried out. Conversely, non-contact techniques such as AS-OCT are non-invasive methods allowing for a quick measurement of a wide number of angle parameters.

All the aforementioned techniques have advantages and disadvantages, summarized in this review, which the clinician should be made aware of, to meaningfully exploit the available resources. Even if a great amount of information and quantitative data on the irido-corneal angle may be achieved, interpretation is not always easy, especially taking into account the lack of normative databases and longitudinal studies [133]. Moreover, the vast majority of studies investigating the diagnostic ability of these instruments has been performed in populations of Chinese ethnicity (Table 2), leaving the question open on how they may perform in mixed-race populations. Finally, none of these methods may be considered a reliable substitute of slit-lamp gonioscopy, that remains the clinical reference standard in the diagnosis and management of narrow angles, according to the clinical guidelines [133].

In more recent years, ACA evaluation has improved with the introduction of deep learning algorithms, potentially valuable in the screening of populations at high risk of primary angle closure, and with poor access to eye care. As a medical specialty that is highly dependent on ancillary imaging tests, the application of artificial intelligence is rapidly increasing in ophthalmology, and the future of deep-learning applications in glaucoma is certainly bright. However, additional studies are required to evaluate the usefulness of the deep learning algorithms in different populations and their applicability to different devices. The importance of telemedicine and the use of virtual ophthalmology has recently also increased, especially in the setting of the so-called COVID-19 era [134]. Gonio-photographs and AS-OCT imaging have been already used for remote evaluation and screening of angle closure, with encouraging results [135]. Although these techniques are likely to require further improvements, they may be considered as auxiliary solutions, in a time of social distancing.

## Figures and Tables

**Table 1:** Main quantitative parameters for the assessment of the anterior segment and the irido-corneal angle by ultrasound biomicroscopy (UBM) and anterior segment optical coherence tomography (OCT).

Parameter	Description	Technology
Anterior chamber depth (ACD)	Distance between the corneal endothelium and the anterior surface of the lens	OCT
Anterior chamber width (ACW)	Distance between the scleral spurs in the nasal and temporal quadrants	OCT
Angle opening distance (AOD)	Distance between the trabecular meshwork and the iris at 500 (AOD 500) or 750 μm (AOD 750), anteriorly to the scleral spur	UBM/OCT
Angle recess area (ARA)	The triangular area (ARA 500 or 750) bounded by the AOD 500 or 750, the anterior iris surface and the inner corneo-scleral wall	OCT
Iris-ciliary process distance (ICPD)	Distance between the iris and the ciliary process along the line of TCPD	UBM
Iris thickness 1 (ID1)	Iris thickness at 500 μm anterior to the scleral spur	UBM
Iris thickness 2 (ID2)	Iris thickness at 2 mm from the iris roof	UBM
Iris thickness 3 (ID3)	Maximum iris thickness near the pupillary edge	UBM
Iris-lens angle (ILAθ)	Angle between the iris and the lens near the pupillary edge	UBM
Iris-lens contact distance (ILCD)	Contact distance between the iris and the lens	UBM
Iris thickness (IT)	Iris thickness measured at 750 um (IT 750) or 2000 μm (IT2000) from the scleral spur	OCT
Iris-zonule distance (IZD)	Distance between the iris and the zonule along the line of TCPD	UBM
Lens vault (LV)	The perpendicular distance between the anterior pole of the lens and the horizontal line joining the 2 scleral spurs on horizontal AS-OCT scans	OCT
Trabecular-ciliary process distance (TCPD)	Distance between the trabecular meshwork and the ciliary process at 500 μm anterior to the scleral spur	UBM
Trabecular-iris angle (TIAθ1)	Angle of the angle recess	UBM
Trabecular iris space area (TISA)	Trapezoidal area (TISA 500 or 750) bounded by the AOD 500 or 750, the anterior iris surface and the inner corneo-scleral wall	OCT

**Table 2:** Area under the curve (AUC) with 95% Confidence Interval (CI) for the detection of angle closure and narrow angle. Data reported by techniques and demographics.

Technique	AUC	95% CI	Main Ethnicity (%)	Mean Age (SD) (y)
**Angle closure detection**				
EyeCam [54]	0.98	0.93–1.00	Chinese (70.4%)	60.7 (12.6)
Manual grading EyeCam [51]	0.88	0.81–0.96	Chinese (72.9%)	60.5 (12.9)
Automated grading EyeCam [51]	0.74	0.63–0.85	Chinese (72.9%)	60.5 (12.9)
Visante AS-OCT [54]	0.85	0.76–0.92	Chinese (70.4%)	60.7 (12.6)
Visante AS-OCT [98]	0.76	0.74–0.78	Chinese (86.8%)	60.8 (6.8)
CASIA SS-1000 SS-OCT [107]	0.84	0.81–0.88	Chinese (87.3%)	61.8 (6.7)
Deep Learning algorithm (ResNet-18) [128]	0.93	0.92–0.94	Chinese (100%)	61.1 (8.1)
**Narrow angle detection**				
Ultrasound Biomicroscopy (ARA 750) [37]	0.97	0.92–1.00	White (58.3%)	42.9 (n/a)
Scheimpflug Photography [84]	0.93	0.90–0.96	Indian (100%)	56.2 (6.5)
Scanning peripheral ACD analyzer [76]	0.79	0.70–0.87	Chinese (94.5%)	65.5 (8.2)
Modified Van Herick [76]	0.87	0.80–0.94	Chinese (94.5%)	65.5 (8.2)

ACD: Anterior chamber depth; ARA: Angle recess area; AS-OCT: Anterior segment OCT; AUC: Area under the curve; SD: Standard deviation; SS-OCT: Swept source OCT.

## Abbreviation

ACA	Anterior Chamber Angle
ACD	Anterior Chamber Depth
ACV	Anterior Chamber Volume
ACW	Anterior Chamber Width
AOD	Angle Opening Distance
ARA	Angle Recess Area
AS-OCT	Anterior Segment Optical Coherence Tomography
AUC	Area Under the Curve
BELL	Band of extra-canalicular limbal lamina
CB	Ciliary Body Band
GPS	Gonio-Photographic Systems
IC	Iris Curvature
ICC	Intraclass correlation coefficient
ICPD	Iris-ciliary process distance
ILAθ	Iris-lens angle
ILCD	Iris-lens contact distance
IOL	Intra-Ocular Lens
IT	Iris thickness
IV	Iris volume
ITC	IridoTrabecular Contact
IZD	Iris-zonule distance
LACDM	Limbal Anterior Chamber Depth Measurement
LV	Lens Vault
PAS	Peripheral Anterior Synechiae
SC	Schlemm’s Canal
SD-OCT	Spectral-Domain Optical Coherence Tomography
SL	Schwalbe’s line
SL-AOD	Schwalbe’s line-angle opening distance
SL-TISA	Schwalbe’s line trabecular-iris space area
SP	Scheimpflug Photography
SPAC	Scanning Peripheral Anterior Chamber depth analyzer
SS	Scleral spur
SS-OCT	Swept-Source Optical Coherence Tomography
TCPD	Trabecular-Ciliary Process Distance
TD-OCT	Time-Domain Optical Coherence Tomography
TIA	Trabecular-Iris Angle
TISA	Trabecular-Iris Space Area
TM	Trabecular Meshwork
UBM	Ultrasound Biomicroscopy
VHG	Van Herick Grading
